# Genomic Understanding of an Infectious Brain Disease from the Desert

**DOI:** 10.1534/g3.117.300421

**Published:** 2018-01-11

**Authors:** Leandro F. Moreno, Abdalla A. O. Ahmed, Balázs Brankovics, Christina A. Cuomo, Steph B. J. Menken, Saad J. Taj-Aldeen, Hani Faidah, J. Benjamin Stielow, Marcus de M. Teixeira, Francesc X. Prenafeta-Boldú, Vania A. Vicente, Sybren de Hoog

**Affiliations:** * Westerdijk Fungal Biodiversity Institute, 3584 CT Utrecht, The Netherlands; † Institute of Biodiversity and Ecosystem Dynamics, University of Amsterdam, 1090 GE, The Netherlands; ‡ Department of Basic Pathology, Federal University of Paraná State, Curitiba, PR, Brazil; § Department of Microbiology, College of Medicine, Umm Al-Qura University, 24381 Makkah, Saudi Arabia; ** Broad Institute of Massachusetts Institute of Technology and Harvard, Cambridge, Massachusetts 02142; †† Microbiology Division, Department of Laboratory Medicine and Pathology, Hamad Medical Corporation, Box 3050, Doha, Qatar; ‡‡ Division of Pathogen Genomics, Translational Genomics Research Institute, Flagstaff, Arizona 86005; §§ GIRO Joint Research Unit IRTA-UPC, Torre Marimon, Caldes de Montbui, E-08140, Barcelona, Catalonia, Spain; *** Center of Expertise in Mycology of Radboudumc/CWZ, Nijmegen, 6525 GA, The Netherlands

**Keywords:** black yeast, comparative genomics, Chaetothyriales, cerebral phaeohyphomycosis

## Abstract

*Rhinocladiella mackenziei* accounts for the majority of fungal brain infections in the Middle East, and is restricted to the arid climate zone between Saudi Arabia and Pakistan. Neurotropic dissemination caused by this fungus has been reported in immunocompromised, but also immunocompetent individuals. If untreated, the infection is fatal. Outside of humans, the environmental niche of *R. mackenziei* is unknown, and the fungus has been only cultured from brain biopsies. In this paper, we describe the whole-genome resequencing of two *R. mackenziei* strains from patients in Saudi Arabia and Qatar. We assessed intraspecies variation and genetic signatures to uncover the genomic basis of the pathogenesis, and potential niche adaptations. We found that the duplicated genes (paralogs) are more susceptible to accumulating significant mutations. Comparative genomics with other filamentous ascomycetes revealed a diverse arsenal of genes likely engaged in pathogenicity, such as the degradation of aromatic compounds and iron acquisition. In addition, intracellular accumulation of trehalose and choline suggests possible adaptations to the conditions of an arid climate region. Specifically, protein family contractions were found, including short-chain dehydrogenase/reductase SDR, the cytochrome P450 (CYP) (E-class), and the G-protein β WD-40 repeat. Gene composition and metabolic potential indicate extremotolerance and hydrocarbon assimilation, suggesting a possible environmental habitat of oil-polluted desert soil.

*Rhinocladiella* is a polyphyletic genus residing in the fungal family Herpotrichiellaceae (order Chaetothyriales), comprising a number of opportunistic pathogens on humans (Arzanlou *et al.* 2007) and having phylogenetic affinity to the so-called black yeasts. *Rhinocladiella mackenziei* is unique within the genus in that it causes primary central nervous system infection in humans, with the brain as yet being the only known habitat of the fungus. Other species such as *R. aquaspersa* and *R. basitona* may cause opportunistic skin infections; however, there is no report of extracutaneous dissemination. *R. mackenziei* is confined to the Middle East and adjacent desert zones, and is especially frequent in Saudi Arabia, Pakistan, and Kuwait (Figure 1 and Supplemental Material, Table S1). Cases reported outside the endemic area, *e.g.*, in Europe or the U.S., have invariably concerned immigrants from countries in the Middle East (Cristini *et al.* 2010).

**Figure 1:**
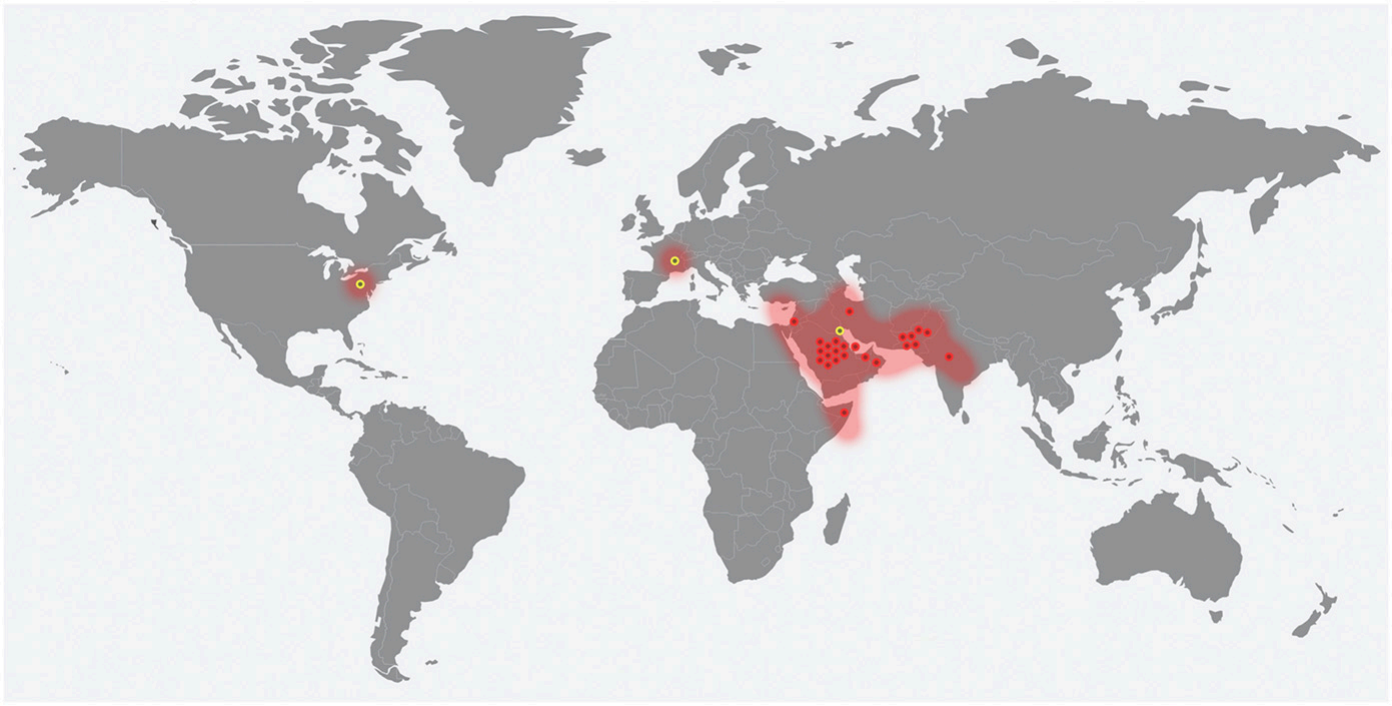
Approximate distribution of 27 cases of cerebral phaeohyphomycosis caused by *R. mackenziei* from 1983 to 2015. Locations were obtained from the literature and are based on the origin of the patient (red circles) and first-admission hospital (yellow circles).

The pathogen probably invades the brain via the blood–brain barrier, presumably after inhalation, but the portal of entry has not been established with certainty (de Hoog *et al.* 2000). The infection mostly affects immunocompromised patients but occasionally immunocompetent individuals are affected, and despite antifungal therapy combined with surgical intervention, a case fatality rate of ≤70% is observed (Li and de Hoog 2009; Badali *et al.* 2010). Neurotropism, defined as the affinity of a pathogen for the central nervous system, is recurrent in the Herpotrichiellaceae, specifically in members of the bantiana and dermatitidis clades (Teixeira *et al.* 2017). Similar to the neurotropic species *Cladophialophora bantiana*, *Fonsecaea monophora*, and *Exophiala dermatitidis*, brain infection by *R. mackenziei* is primary, *i.e.*, the first symptoms of the disease are of a neurological nature (Horré and de Hoog 1999). In this respect, the infection deviates from classical rhinocerebral infection by Mucorales and from orbital skull-base infection by *Aspergillus*, where the brain infection is secondary, having a portal of entry in the sinus and later enters the brain gray matter. Pathogenicity of members of Herpotrichiellaceae is partially explained by general virulence factors such as cell wall melanin (Paolo *et al.* 2006; Seyedmousavi *et al.* 2014) and thermotolerance, which are shared by numerous black fungi, while the assimilation of alkylbenzene hydrocarbons, which are structurally similar to neurotransmitters, has been suggested to be specific to this family (Prenafeta-Boldú *et al.* 2006). Although the exact mechanism by which melanin can enhance the virulence in pathogenic fungi remains unclear, recent literature argues that the pigmentation of the cell wall might play a protective role in fungal cells against free radicals, enzymatic or microbial lysis, and extreme temperatures (Jacobson 2000).

Since *R. mackenziei* thus far has not been isolated from any environmental source, its natural reservoir and route of infection remain unclear. Nevertheless, the presence of genes that could confer tolerance against environmental stressors has been reported in *R. mackenziei* (Teixeira *et al.* 2017), indicating that this fungus might reside under the conditions of heat and dryness of arid climates. Genome content determination and comparative genomics with closely related species may further our understanding of metabolic adaptations in *R. mackenziei* with clues to its potential natural habitat. Black yeasts in general display remarkably diverse lifestyles, with a predilection for extreme and toxic environments such as those rich in aromatic compounds or heavy metals, or with high temperatures, increased salinity, and scarcity of nutrients (Paolo *et al.* 2006; Prenafeta-Boldú *et al.* 2006; Zhao *et al.* 2010). A recent comparative genomic analysis of 23 black yeast species (Teixeira *et al.* 2017), including the type strain of *R. mackenziei*, CBS 650.93, revealed a wide diversity of mechanisms associated with nutrient acquisition. These oligotrophic fungi exhibit specific gene family expansions, including alcohol (ADH) and aldehyde dehydrogenases (ALDHs), membrane transport proteins, and a diverse ensemble of CYPs, which are believed to be essential for extremotolerance and survival of environmental stress conditions (Teixeira *et al.* 2017). On the other hand, gene family contractions have not been reported in black yeasts (Z. Chen *et al.* 2014). The *R. mackenziei* genome harbors a large number of gene clusters involved in secondary metabolite production, genes encoding proteolytic and carbohydrate-active enzymes, and a wide set of proteins involved in melanin production through distinctive pathways (Teixeira *et al.* 2017).

In the present study, we performed the whole-genome resequencing of two additional *R. mackenziei* strains isolated from brain biopsies of patients from Saudi Arabia and Qatar. Subsequent variant calling analysis provided a catalog of sequence variations and their putative biological consequences. In addition, we compared the *R. mackenziei* genomes to other closely related fungi in order to determine genetic signatures such as species-specific genes. Using Fisher’s exact test combined with *q*-value correction, we confirmed gene family expansions, but also noted gene family contractions shared by *R. mackenziei* and other neurotropic fungi. GO enrichment analyses were used to predict gene functions of species-specific genes, paralogs, and genes shared with other neurotropic black yeasts. A survey of potential pathogenicity-related genes present in *R. mackenziei* was carried out comparing its proteome against a curated database.

## Materials and Methods

### Strains and DNA extraction

To extract genomic DNA, fungal mycelia of the *R. mackenziei* strains IHM 22877 and dH24460 were harvested from fresh cultures on Sabouraud’s Glucose Agar, washed using sterile Tris-EDTA buffer (TE), pH 8.0 in 2 ml screw-capped tubes, and then resuspended in 500 µl TE buffer. Fungal cell walls were disrupted using 0.5 mm glass beads in a BioSpec Mini-Beadbeater-16 (BioSpec) for 5 min and cooled on ice for an additional 5 min. DNA solutions were separated using two phenol/chloroform (1:24, pH 8.0) extractions. DNA was then precipitated by isopropanol, washed with 70% ethanol, dried at room temperature, and resuspended in 35 µl TE buffer, pH 8.0. DNA quantity and quality were determined using Qubit (Invitrogen, Applied BioSystems), and an Agilent Bio Analyzer 2100 using a 1000 DNA Chip (Agilent).

### Genome sequencing and de novo assembly

For genome sequencing, the libraries were constructed using an Illumina NexteraXT Library Preparation Kit and samples were barcoded using a NexteraXT Index Kit (Illumina). The libraries were validated and quantified without bead normalization using an Agilent Bio Analyzer 2100 1000 DNA Chip (Agilent). The dH24460 and IHM 22877 genomes were sequenced on the Illumina MiSeq platform using a paired-ends protocol and a version-3 600 cycles kit. Quality control was performed using FastQC v0.11.3 (http://www.bioinformatics.bbsrc.ac.uk/projects/fastqc) and low-quality sequences were removed by Trimmomatic (Leading: 3, Trailing: 3, Slidingwindow: 4:15) (Bolger *et al.* 2014). High-quality reads were assembled *de novo* using SPAdes v3.6.2 (Bankevich *et al.* 2012).

### Read mapping and SNP calling

For SNP calling, high-quality reads were mapped against the reference genome of the type strain *R. mackenziei* CBS 650.93 (accession JYBU00000000) by using Burrows Wheeler Aligner (BWA) v0.7.5 mem (Li and Durbin 2009). The reference genome assembly consisted of 32.47 Mb with a GC content of 50.42%, and was organized in 130 contigs linked by paired-end reads into 17 scaffolds. Alignments were improved by GATK RealignerTargetCreator and IndelRealigner realigning reads around insertion/deletion (indels) in order to minimize bases mismatching the reference (GATK v. 3.4–46) (Van der Auwera *et al.* 2013). Variants were identified with GATK haplotypecaller v. 3.4–46. Hard filters were applied using the GATK VariantFiltration according to GATK best-practices v.3 guidelines [QD < 2.0, MQ < 40.0, FS > 60.0, HaplotypeScore > (average value + 2*SD), MQRankSum < −12.5, ReadPosRankSum < −8.0]. In addition, SNPs and indels were called with Pilon version 1.4 (Walker *et al.* 2014) using default settings. Predictions were combined using the tools SelectVariants and CombineVariants (https://software.broadinstitute.org/gatk/). SNP annotation was performed by VCFannotator (http://vcfannotator.sourceforge.net/) to assess whether the SNP was found within an untranslated region, intron, or coding exon. Mutations were classified into synonymous (SYN), nonsynonymous (NSY), read-through (RTH), and nonsense (STP). We used the alignment-based method PROVEAN to predict the effect of NSY SNPs on protein function (Choi *et al.* 2012). For this purpose, proteins carrying single-amino acid substitutions were compared to their homologs in the NCBI nonredundant protein database, and the substitution frequency and chemical properties of the changed amino acids were taken into account to estimate a score that was used to measure the effect of the variation (Choi *et al.* 2012).

### Structural variation (SV) identification

To detect genomic SVs from high-throughput sequencing data, the *R. mackenziei* strains IHM 22877 and dH24460 were analyzed combining two different methods. First, we ran Breakdancer (Chen *et al.* 2009) to predict SV based on paired-end read mapping (default parameters). To increase the sensitivity and specificity of the call, we used the output of Breakdancer as input for Pindel (Ye *et al.* 2009), which employs split-read alignments to detect SV, setting the options –b and –M 10 as described elsewhere (Ghoneim *et al.* 2014).

### Protein annotation of strains IHM 22877 and dH24460

To determine the protein sequences corresponding to the newly sequenced strains, 11,382 proteins of the type strain *R. mackenziei* CBS 650.93 (JYBU00000000) were aligned to the genome of the strains IHM 22877 and dH24460 using the homology-based predictor Exonerate v. 2.2.0 (Slater and Birney 2005) with the parameters–model protein2genome–percentage 90. Pathway and KOG prediction were assessed using KAAS (bidirectional best hit) (Moriya *et al.* 2007). Putative secreted proteins were identified using SignalP 4.1 (Petersen *et al.* 2011) and WoLF PSORT 0.2 (Horton *et al.* 2007). Secreted proteins were assessed for the presence of transmembrane helices by Phobius (Kall *et al.* 2007) and THMMH 2.0 (Sonnhammer *et al.* 1998). CYPs were predicted as described elsewhere (Teixeira *et al.* 2017). CYP proteins that could not be assigned to families or subfamilies based on the International P450 Nomenclature Committee were aligned and subjected to phylogenetic analyses, as described elsewhere (W. Chen *et al.* 2014). Pathogenicity-related genes were identified by means of BLASTP searches (identity > 50%) against the curated PHI-database version 4.0 (Winnenburg *et al.* 2006).

### Ortholog detection

Orthology data set was generated clustering the *R. mackenziei* proteins with 20 previously sequenced black yeast species (*E. xenobiotica* CBS 118157, *E. aquamarina* CBS 119918, *E. mesophila* CBS 402.95, *E. spinifera* CBS 899.68, *E. oligosperma* CBS 725.88, *E. sideris* CBS 121828, *E. dermatitidis* NIH 8656, *Capronia epimyces* CBS 606.96, *Ca. coronata* CBS 617.96, *Ca. semiimmersa* CBS 27337, *C. carrionii* CBS 160.54, *C. immunda* CBS 834.96, *C. bantiana* CBS 173.52, *C. psammophila* CBS 110553, *C. yegresii* CBS 114405, *F. pedrosoi* CBS 271.37, *F. multimorphosa* CBS 102226, *Phialophora europaea* CBS 101466, *P. attae* CBS 131958, *Coniosporium apollinis* CBS 100218, and *Verruconis gallopava* CBS 437.64). Orthologs and paralogs were determined by means of OrthoMCL (Li *et al.* 2003), with a Markov inflation of 1.5 and a maximum *e*-value of 10^−5^. Species-specific proteins were extracted using a custom Bash script.

### Mitochondrial genome assembly and annotation

Mitochondrial genome sequences of the strains were assembled using GRAbB (Brankovics *et al.* 2016) (https://github.com/b-brankovics/grabb) from the raw sequencing data. The bait used for the GRAbB assembly was the mitochondrial genome of *E. dermatitidis* (NW_008751656). Initial mitochondrial genome annotations were done using MFannot (http://megasun.bch.umontreal.ca/cgi-bin/mfannot/mfannotInterface.pl) and manually adjusted. Annotation of tRNA genes was improved using tRNAscan-SE (Pavesi *et al.* 1994). Annotation of protein-coding genes was corrected by aligning intronless homologs to the genome.

### Data availability

The sequencing data from this study have been submitted to the GenBank: accession numbers GCA_001723215 (strain IHM 22877) and GCA_001723235 (strain dH24460). Raw sequence data can be accessed through the NCBI Sequence Read Archive: accession number SRP128022. Table S1 summarizes the distribution of 27 cases of cerebral phaeohyphomycosis caused by *R. mackenziei*. Table S2 contains genes with significant mutations according to PROVEAN. Table S3 and Table S4 show the expanded GO categories between the neurotropic species. Table S5 contains the expanded GO categories in paralog genes of *R. mackenziei*. Table S6 contains the singleton proteins found in *R. mackenziei*. Table S7 contains the *R. mackenziei* secretome. Table S8 contains the CYP in *R. mackenziei*. Table S9 shows the gene family expansions and contractions observed in *R. mackenziei*. Table S10 contains the pathogenicity related genes. Figure S1 shows the frequency and size of indels in coding and noncoding regions.

## Results

### Sequencing and mapping

**Figure 2:**
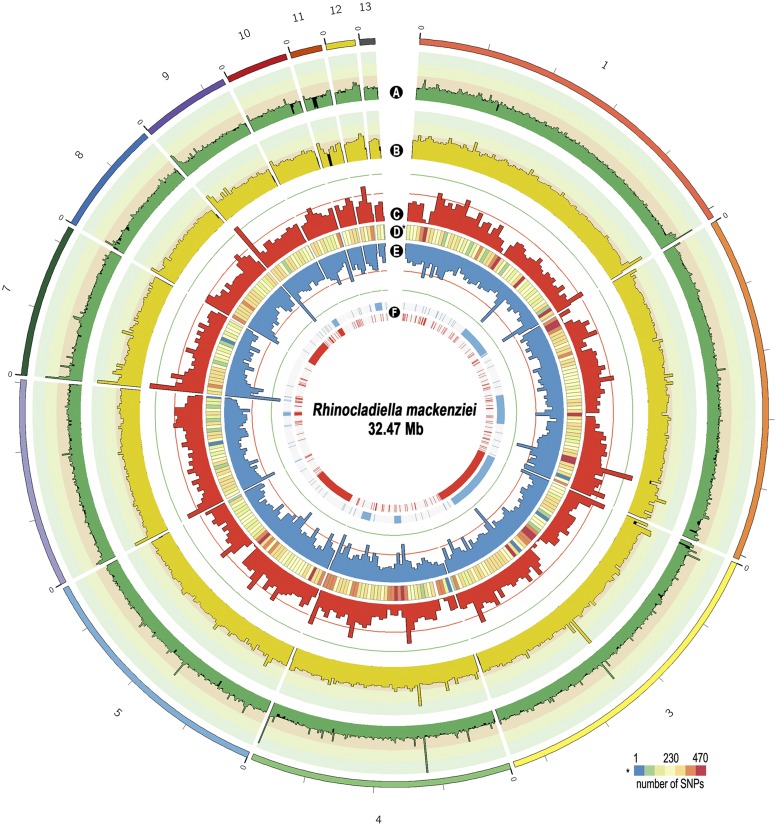
Overview of the 13 largest scaffolds of *R. mackenziei* strains dH24460 and IHM 22877. From outside to inside the ring: (A) read coverage strain dH24460, (B) read coverage strain dH24460, (C) single nucleotide polymorphism (SNP) density for the strain dH24460, (D) difference in the SNP density between the two strains compared to the reference, (E) SNP density for the strain IHM 22877, and (F) distribution of potential inversions along the genomes of dH24460 (red) and IHM 22877 (blue).

To characterize variants of two *R. mackenziei* strains from Qatar and Saudi Arabia compared to the reference genome, each strain was deeply sequenced using Illumina MiSeq. After filtering, a total of 12,996,368 and 10,131,024 high-quality reads were generated for the strains dH24460 and IHM22877, respectively. Among these reads, 11,542,511 and 9,568,960 reads were mapped to the *R. mackenziei* CBS 650.93 reference genome (Figure 2).

Sequencing of both dH24460 and IHM 22877 strains did not produce reads corresponding to the region of scaffold 15 in the reference genome. This scaffold was 4775 bp long and contained two protein-coding genes (Z518_11416 and Z518_11417) annotated as hypothetical proteins, holding PFAM functional domains associated with LTR retrotransposons (PF03732)/zinc finger domains (PF00098) and manipulation of chromatin (PF00385). In order to further investigate the genomic composition of scaffold 15, we used TransposonPSI (http://transposonpsi.sourceforge.net) to identify potential transposable elements. The scaffold harbored three putative LTR retroelements: one classified as Ty1/Copia and two classified as gypsy.

### Variant calling

**Figure 3:**
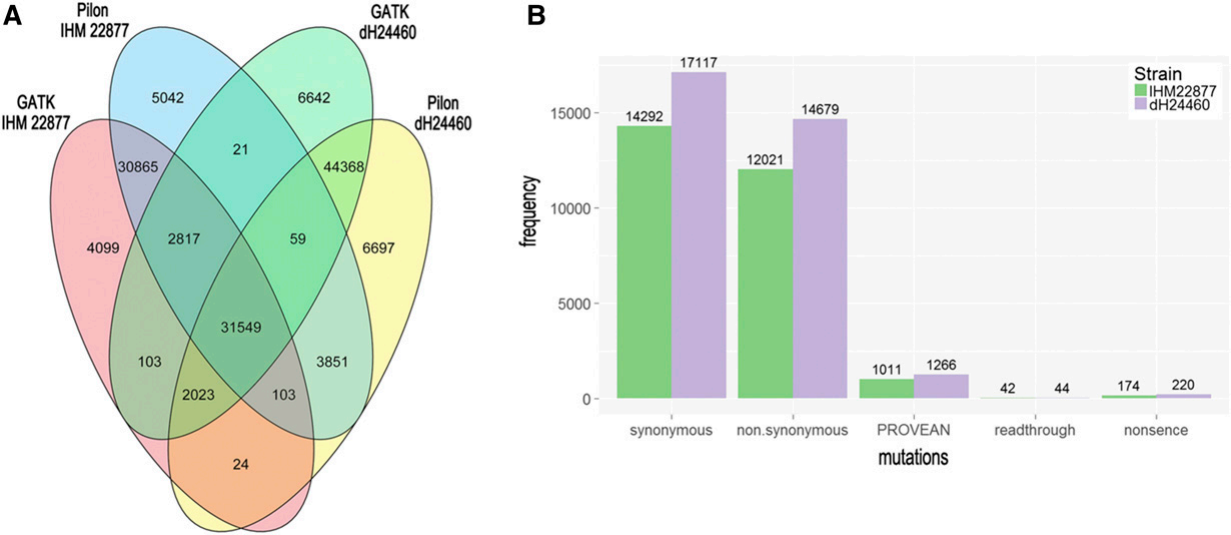
(A) Intersection of the two single nucleotide polymorphism (SNP) calling analyses. (B) Total number of SNPs in different categories according to VCFannotator and the number of proteins impacted by nonsynonymous mutations on the biological function assessed using the tool PROVEAN.

The total number of genomic variations relative to the reference genome was detected in the resequenced *R. mackenziei* strains using two methods. The intersection of the two SNP calling analyses is represented in Figure 3A and calls made by both programs were used for further analyses.

Genome-wide variation identification revealed that the strains IHM 22877 and dH24460 contain 65,333 and 77,999 SNPs, respectively, compared to the reference. The highest concentration of SNPs was found in scaffold 11 with, on average, a SNP every 294 bp for IHM 22877 and one every 342 bp for strain dH24460. The lowest density of SNPs was found in scaffold 13 (one every 426 bp on average) for strain dH24460 and in scaffold 3 (one every 484 bp on average) for strain IHM 22877. The distribution of SNPs along the genomes is shown in Figure 2C and E. Regions of high SNP density were located near ends of the scaffolds 1, 4, 6, 7, 8, 9, 10, and 11, suggesting that these regions might correspond to subtelomeres, shown to be hypervariable regions in other studies, potentially undergoing higher rates of mutation (Cuomo *et al.* 2007).

In order to obtain a comprehensive variant annotation, VCFannotator (see *Materials and Methods*) was used to classify the mutations into four distinctive categories: SYN, NSY, RTH, and STP. Variant annotation showed that 78,041 SNPs (54.4%) were located in intergenic regions (dH24460: 42,249 and IHM 22877: 35,792), while 65,291 SNPs (45.6%) were located in intronic (dH24460: 3,757 and IHM 22877: 3080) and exonic (dH24460: 31,993 and IHM 22877: 26,461) regions. Of the total SNPs localized in coding regions, 31,409 were SYN (dH24460: 17,117 and IHM 22877: 14,292), 26,700 were NSY (dH24460: 14,679 and IHM 22877: 12,021), 86 were RTH (dH24460: 44; IHM 22877: 42), and 394 were STP (dH24460: 220; IHM 22877: 174) (Figure 3B).

Leveraging published genomes, we identified 2468 single-copy orthologs (SCOs) that were common to 22 black yeasts (see *Materials and Methods*). Among this gene set, 325 and 422 intronic SNPs, and 5287 (3258 SYN and 2024 NSY) and 6547 (2577 SYN and 3964 NSY) exonic SNPs, were identified in the strains IHM 22877 and dH24460, respectively. SNPs within SCO exons included only four STP (1%) and RTH mutations of stop codons (8%) (Table 1). STP mutation promotes early translational termination of messenger RNAs, resulting in truncated proteins and with possible deleterious effect on the protein function. As expected, the low frequency of mutations in SCO genes confirms the high level of conservation of this gene set in black yeasts.

**Table 1:** SCO genes containing STP and RTH suppression mutations

Strain	Gene ID	Annotation	Mutation	Category
IHM 22877	Z518_03085	Protein kinase-like	Codon: Gaa → Taa	STP mutation
IHM 22878	Z518_11082	Protein of unknown function	Codon: taT → taG	STP mutation
IHM 22879	Z518_05460	Protein of unknown function	Codon: tGg → tAg	STP mutation
IHM 22880	Z518_01487	Protein of unknown function	Codon: taT → taG	RTH suppression
IHM 22881	Z518_05409	Ribonuclease	Codon: Tag → Gag	RTH suppression
dH24460	Z518_11082	Protein of unknown function	Codon: taT → taG	STP mutation
dH24460	Z518_10302	Protein of unknown function	Codon: Tga → Cga	RTH suppression
dH24460	Z518_06350	Histidine phosphatase superfamily	Codon: Tga → Cga	RTH suppression
dH24461	Z518_09833	GTP cyclohydrolase	Codon: Tga → Cga	RTH suppression
dH24462	Z518_04886	Protein of unknown function	Codon: Tga → Cga	RTH suppression
dH24463	Z518_03836	Protein of unknown function	Codon: Tga → Cga	RTH suppression

ID, identifier; STP, nonsense; RTH, read-through.

### Functional effect of SNPs on proteins

Among the NSY SNPs, 1266 (8.5%) and 1011 (8.3%) mutations in strains dH24460 and IHM 22877, respectively, were considered to have significant effects on protein function (Table S2). The proteins carrying these mutations, in both strains, were enriched for GO categories involved in oxidoreductase activity (Table 2), including several proteins annotated as CYPs (PF00067), alcohol dehydrogenases (ADHs) (PF08240), and ALDHs (PF00171). Furthermore, we found that paralogous genes, including several CYPs, are enriched for such mutations that can affect their properties (*P* < 2.2 × 10^−16^), which may contribute to diversification of these functions. Among the 872 putative paralogous genes predicted in *R. mackenziei*, 186 carry at least one severe mutation.

**Table 2:** GO categories enriched among genes with NSY SNPs and scored by PROVEAN as severe mutations

*R. mackenziei* Strain	GO Category	Term	*P*-Value	Top Five PFAM Domains
IHM 22877	GO:0016491	Oxidoreductase activity	1.74E−08	PF00106, PF00107, PF08240, PF01266, PF00171
	GO:0055114	Oxidation-reduction process	3.00E−08	PF00067, PF08240, PF00107, PF01266, PF00732
	GO:0042623	ATPase activity, coupled	5.30E−08	
	GO:0016887	ATPase activity	7.13E−08	PF00005
	GO:0015399	Primary active transmembrane transporter activity	7.90E−07	
	GO:0015405	P-P-bond-hydrolysis-driven transmembrane transporter activity	7.90E−07	
	GO:0043492	ATPase activity, coupled to movement of substances	1.37E−06	
	GO:0042626	ATPase activity, coupled to transmembrane movement of substances	2.08E−06	PF00664, PF06422
	GO:0016462	Pyrophosphatase activity	2.43E−06	
	GO:0016818	Hydrolase activity, acting on acid anhydrides, in phosphorus-containing anhydrides	4.52E−06	
	GO:0016820	Hydrolase activity, acting on acid anhydrides, catalyzing transmembrane movement of substances	4.57E−06	
	GO:0017111	Nucleoside-triphosphatase activity	5.58E−06	
	GO:0016817	Hydrolase activity, acting on acid anhydrides	9.17E−06	
	GO:0003824	Catalytic activity	2.29E−05	PF00501, PF01048, PF02515, PF00128, PF01557
	GO:0022804	Active transmembrane transporter activity	5.85E−05	
	GO:0046906	Tetrapyrrole binding	1.30E−04	
	GO:0020037	Heme binding	1.30E−04	PF00067, PF01077, PF00199
	GO:0016705	Oxidoreductase activity, acting on paired donors, with incorporation or reduction of molecular oxygen	1.59E−04	PF00067
dH24460	GO:0016491	Oxidoreductase activity	3.11E−07	PF00106, PF00107, PF08240, PF02668, PF01565
	GO:0055114	Oxidation–reduction process	1.45E−06	PF00067, PF08240, PF00107, PF02668, PF00732
	GO:0016705	Oxidoreductase activity, acting on paired donors, with incorporation or reduction of molecular oxygen	9.97E−06	PF00067
	GO:0005506	Iron ion binding	4.32E−05	PF00067, PF10637, PF00848

GO, gene ontology; PFAM, protein family database.

### Indel variation

We identified 26,754 potential indels (dH24460: 14,837 and IHM 22877: 11,917). Overall, 7.7% of these mutations were found in coding regions with a mean length of 7.3 and 8.4 bp for insertions, and 14.9 and 16.6 bp for deletions, in the strains dH24460 and IHM 22877, respectively. Indels that are multiples of 3 bp, and thus cannot cause frameshifts, account for 42% of the coding indels in dH24460 and 38% in IHM 22877 (Figure S1). Intronic regions comprise 5.8% of the indels (dH24460: 845 and IHM 22877: 722). Rates of SNPs and indels were more frequent in *R. mackenziei* dH24460, revealing that this is more highly diverged from the reference isolate.

Overall, *R. mackenziei* strain dH24460 possessed 95 potential inversions with a mean length of 136,002 bp and strain IHM 22877 harbored 287 potential inversions with a mean length of 45,324 bp (Figure 2F). The three longest inversions were identified in scaffold 3 corresponding to 64.5 and 60.3% of the total length of inversions in strains IHM 22877 and dH24460, respectively. The highest density of inversions was found in scaffold 1 in both strains of *R. mackenziei* (Figure 2).

### Protein-coding gene annotation and general features

Protein-coding genes were predicted based on alignments of proteins of *R. mackenziei* CBS 650.93 against the genome of the strains IHM 22877 (GenBank assembly accession: GCA_001723215) and dH24460 (GenBank assembly accession: GCA_001723235). The reference protein set contained 11,382 proteins. In total, 11,148 genes were predicted in strain IHM 22877 and 11,191 genes in dH24460, covering 97.9 and 98.2% of the gene content of the type strain, respectively. Using the reference protein set, a total of 6349 proteins (55.8%) were assigned to GO terms and 8836 proteins (77.5%) contained a protein family domain. The top five most abundant PFAM domains included WD40 repeat PF00400, membrane transport proteins major facilitator type (MFS) PF07690, fungal transcription factor PF04082, transcription factor type Zn2Cys6 PF00172, and CYP PF00067. Comparative analyses with other black yeasts showed that the *R. mackenziei* genomes contained a large number of highly conserved homologs. Best-hits BLASTP (cut-off identity > 90%) were found against the saprobic species *E. xenobiotica* (10.3%), *F. multimorphosa* (9.7%), and *C. immunda* (8.6%), followed by two neurotropic fungi, *E. dermatitidis* (8.4%) and *C. bantiana* (8.0%). The lowest similarities were found with *P. attae* (0.5%) and *P. europaea* (0.4%), members of black yeast family Cyphellophoraceae. Interestingly, highly conserved homologs shared between the neurotropic fungi *R. mackenziei*, *C. bantiana*, and *E. dermatitidis*, spanned across several GO categories (Table S3 and Table S4), including organonitrogen compound biosynthetic/metabolic process (GO:1901566, GO:1901564), amide biosynthetic process (GO:0043604), and peptide metabolic process (GO:0006518).

Orthology analysis using Orthomcl determined the protein families conserved in *R. mackenziei* and other 22 black yeast species. This analysis indicated the presence of 872 putative paralogs in *R. mackenziei*. These genes belong to the same GO categories described above. Additional mechanisms involving transmembrane transport, mainly of nitrogen, were found overrepresented in *R. mackenziei*, such as nitrogen transport (GO:0071705), amino acid transport (GO:0006865, GO:0003333, and GO:0015171), peptide transport (GO:0015833), amide transport (GO:0042886), and amino sugar and aminoglycan biosynthetic processes (GO:0046349) (Table S5). These findings raise new questions regarding the potential involvement of nitrogen-containing compounds and neurotropism observed in some black yeast species.

### Singleton proteins

*R. mackenziei* possesses 755 orphan proteins that are unique among the black yeasts according to Orthomcl (see *Materials and Methods*), and therefore were not included in any group of homologs (Table S6). InterProScan searches performed on this data set showed that 241 proteins possess conserved functional domains. The top five most frequent Interpro protein domains (IPRs) in orphan proteins include IPR020683 (ankyrin repeat-containing domain), IPR001128 (CYP), IPR011701 (MFS transporters), IPR010730 (heterokaryon incompatibility), and IPR002347 (short-chain dehydrogenase/reductase). Despite the low sequence conservation of these proteins among black yeasts, BlastP analysis against the NCBI database revealed that many of these enzymes are often found in plant-associated fungi (Table S6), for example the putative glutathione S-transferase protein Z518_06694 (86% identity with *Eutypa lata*), nitrilase Z518_09980 (66% identity with *Botrytis cinerea*), CYP Z518_01777 (93% identity with *Oidiodendron maius*), the putative siderophore iron transporter Z518_06150 (identity 77% with *Phaeomoniella chlamydospora*), and the myo-inositol transporter Z518_03615 (74% with *O. maius*). Other enzymes involved in the iron transport, such as the siderophore iron transporters Z518_10557 and Z518_10557, were similar to the saprobic fungus *Purpureocillium lilacinum*.

### R. mackenziei secretome

In *R. mackenziei*, the pool of secreted proteins (secretome) includes several virulence factors, such as proteases and chitinases. Analyses of secretion signal peptides, the N-terminal regions that control the entry of proteins to the secretory pathway, revealed the presence of 216 putative secretory proteins in this fungus. Among these enzymes, 20 peptidases, 46 carbohydrate-active enzymes (CAZY), four laccases, and one CYP were identified (Table S7). The signal peptide was also found in three out of four tyrosinases reported in *R. mackenziei*. Interestingly, extracellular tyrosinases and laccases are involved in oxidation and scavenging of phenolic compounds, as well as in the melanin biosynthesis (van Gelder *et al.* 1997; Li *et al.* 2016).

### CYPs

**Figure 4:**
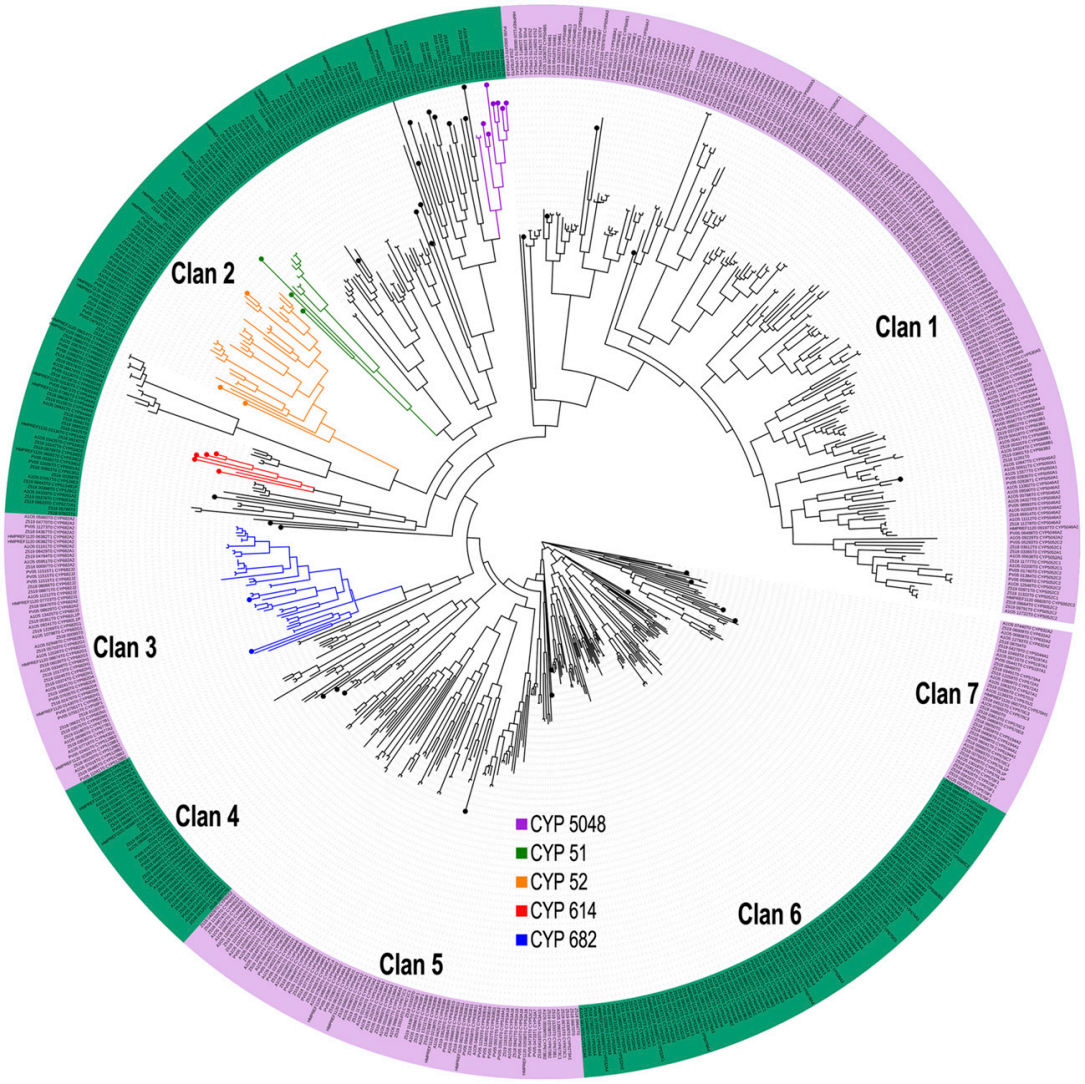
Phylogenetic analysis of cytochrome P450s in black yeasts. The phylogenetic tree was built using 645 P450 protein sequences from *C. bantiana*, *C. psammophila*, *E. dermatitidis*, *E. xenobiotica*, and *R. mackenziei*. The 50 cytochrome P450s used in this study are represented by circles at the ends of the branches.

CYPs are involved in primary, secondary, and xenobiotic metabolism, playing a diverse role in fungal pathogenicity and detoxification/degradation of toxic exogenous compounds. These enzymes may allow the fungus to grow under different stressful conditions or adapt to polluted environments (Crešnar and Petrič 2011). Black yeasts possess one of the highest rates of CYP genes in Ascomycota reported to date (Teixeira *et al.* 2017). These include families of CYPs thought to be involved in the metabolism of phenolic compounds and aromatic hydrocarbons, such as CYP530, CYP682, CYP504, and CYP52 (Teixeira *et al.* 2017). In this study, we analyzed 50 CYPs previously reported in *R. mackenziei* in which their precise families or subfamilies could not be assigned based on the fungal P450 CYPs database (Nelson 1998) (http://blast.uthsc.edu) and following the International P450 Nomenclature Committee rule, which suggests that proteins with > 40% identity and > 55% identity may be clustered under the same family and subfamily, respectively. For that, we used phylogenetic analysis including 645 CYP protein sequences from the black yeasts *C. bantiana*, *C. psammophila*, *E. dermatitidis*, *E. xenobiotica*, and *R. mackenziei* (Teixeira *et al.* 2017). CYPs within the same family/subfamily clustered together in the tree, indicating that their annotation based on the BLASTP identity was correct. Among the 50 unannotated P450s, 33 were accommodated in 18 families with CYPs of other black yeasts, possibly with overlapping functions (Table S8). Three CYPs belonged to the CYP52 family, which is involved in alkane assimilation and fatty acid metabolism in yeasts (Seghezzi *et al.* 1992; Ohkuma *et al.* 1995). Consistent with other studies (Pedrini *et al.* 2013), on the phylogenetic tree, the CYP52 family is very close to the CYP584 family, indicating that these CYPs derived from a single ancestor. Seventeen CYPs were not assigned to any family and may consist of new P450s, specific to *R. mackenziei*, and their accurate nomenclature remains unclear. We identified seven clans (Figure 4), a higher level of P450 classification comprising evolutionarily related families that probably have common functions (Nelson 1998). Clan 1 was the most populated, although the majority of unannotated CYPs in *R. mackenziei* belonged to clan 2.

### Metabolism of fatty acids and two-carbon compounds

*R. mackenziei* harbored genes for the glyoxylate cycle, which allows the assimilation of fatty acids and two-carbon compounds, such as acetate and ethanol, to produce glucose (Kornberg 1966). This pathway has been associated with fungal virulence, as it permits energy production in environments that are poor in complex carbon compounds (Lorenz and Fink 2001). Two enzymes are considered key in the glyoxylate cycle and were identified in *R. mackenziei*: isocitrate lyase (Z518_04722 and Z518_01820) and malate synthase (Z518_01715). Similarly to other black yeast species, *R. mackenziei* possessed a paralog copy for isocitrate lyase.

### Metabolism of aromatic compounds

**Figure 5:**
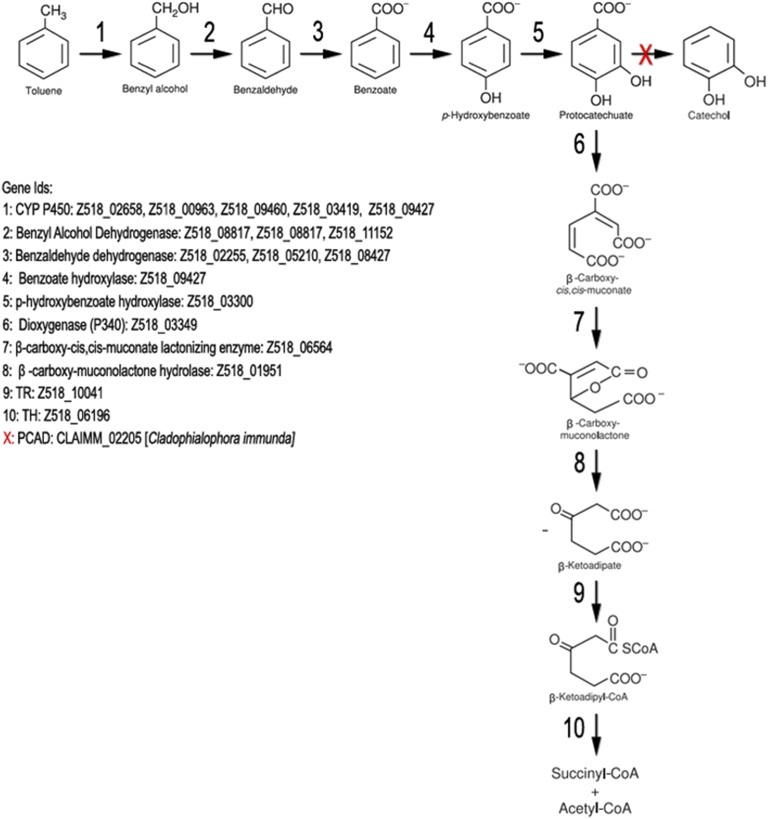
Proposed pathway for toluene metabolism in *R. mackenziei*. Adapted from Parales *et al.* (2008). CYP, cytochrome.

The genome of *R. mackenziei* CBS 650.93 was assessed in order to identify genes linked to the degradation of aromatic compounds, such as benzene, toluene, ethylbenzene, xylene, and styrene, important pollutants from petrochemical and chemical industries. Toluene is initially oxidized to benzyl alcohol by a membrane-bound CYP in toluene-growing cells of the closely related *C. saturnica* CBS 114326, previously confused with *Cladosporium sphaerospermum* (Luykx *et al.* 2003). Comparative analysis against the aromatic hydrocarbon-degrading fungus *C. immunda* revealed the presence of orthologs that resemble the published fungal toluene degradation pathway via protocatechuate (Parales *et al.* 2008; Blasi *et al.* 2017). *R. mackenziei* possesses five highly conserved homologs of CYP that were overexpressed when *C. immunda* was grown in the presence of toluene (Figure 5) (Blasi *et al.* 2017). Among them, the CYP (Z518_09427) belongs to the cytochrome family CYP53, a benzoate para-hydroxylase that is associated with the detoxification of xenobiotic compounds in other ascomycetes (Faber *et al.* 2001; Jawallapersand *et al.* 2014). Furthermore, CYP53 has been proposed as a novel alternative antifungal drug target (Podobnik *et al.* 2008). Benzyl alcohol is converted to protocatechuate through a multistep reaction involving four enzymes (Figure 5: enzymes two–five). Contrary to *C. immunda*, where protocatechuate can be converted to catechol, in *R. mackenziei* the only route for the processing of this compound seems to be via the β-ketoadipate pathway (Figure 5). *R. mackenziei* also possesses enzymes of the styrene and the phenylalanine degradation pathways. Accordingly, styrene might be degraded via phenylacetic acid, which is converted to homogentisate, by a phenylacetate 2-hydroxylase (Z518_00050, Z518_05387, Z518_05992, and Z518_07177), a CYP belonging to the family CYP504. Homogentisate is oxidized to 4-maleylacetoacetate by the homogentisate 1,2-dioxygenase (Z518_05388, Z518_05995, Z518_06895, and Z518_09726), which is converted to fumarylacetoacetate and to fumarate/acetoacetate by the enzymes maleylacetoacetate isomerase (Z518_00072) and fumarylacetoacetase (Z518_00072), respectively. This data suggests that the catabolism of aromatic amino acids, such as phenylalanine, can occur via oxidation to phenylacetate. Previously studies suggested that a CYP504 family member is involved in the catabolism of both phenylacetate and 2-hydroxyphenylacetate acid, which is then oxidized to homogentisate and degraded to fumarate and acetoacetate (Rodriguez-Saiz *et al.* 2001). These pathways may be important to allow this fungus to thrive on environmental contaminants conferring both metabolic versatility and defense against toxic chemicals.

### Iron acquisition strategies

Different strategies for iron acquisition were also present in *R. mackenziei*, allowing the uptake and storage of iron from the host during infection. The enzymes that are required for the production of siderophores (iron-specific chelators) in *E. dermatitidis* were also found in *R. mackenziei*. In contrast to other neurotropic fungi (Z. Chen *et al.* 2014), the *SidD* and *SidF* genes (Z518_02441 and Z518_02442, respectively) are adjacent in *R. mackenziei*. The *SidA* and *SidC* genes (Z518_09385 and Z518_09384, respectively) are adjacent in both *R. mackenziei* and *E. dermatitidis*. The cluster containing the high-affinity iron permease FtrA and the ferroxidase FetC are duplicated in *R. mackenziei* (Z518_02600- Z518_06281 and Z518_02599-Z518_06280), as well as in the neurotropic black fungi *E. dermatitidis* (HMPREF1120_01590-HMPREF1120_04510 and HMPREF1120_01589-HMPREF1120_04509) and *C. bantiana* (Z519_09337- Z519_11106 and Z519_09338-Z519_1110).

### Mechanisms of osmotolerance

*R. mackenziei* possesses distinctive mechanisms to enable its survival under extreme environmental conditions, such as the heat and the dryness of the desert where the environmental phase of the fungus is thought to reside. To avoid dehydration of cells, some fungi are able to accumulate neutral and low-molecular-weight compounds intracellularly, for example the disaccharide trehalose and the quaternary ammonium compound choline (Nikawa *et al.* 1990; Blomberg and Adler 1992; Park and Gander 1998). *R. mackenziei* possesses a putative choline permease (Z518_09106, TCDB family 2.A.3.4.1) that may be involved in extracellular choline uptake, which is subsequently converted to the osmoprotectant glycine betaine by the enzymes choline dehydrogenase (Z518_00519) and betaine-ALDH (Z518_07684). Among the black yeasts, homologs of choline permease are only found in the hydrocarbon-degrading fungus *E. oligosperma* (PV06_02302) and in the plant-associated fungus *Ca. epimyces* (A1O3_02156). In addition, we found that *R. mackenziei* harbors duplicated enzymes that catalyze the biosynthesis of trehalose through the processing of α-D-glucose-1P: the trehalose 6-phosphate synthase (Z518_04836 and Z518_10013) and the trehalose 6-phosphate phosphatase (Z518_02299 and Z518_05120).

### Gene family expansions and contractions

To investigate the gain and loss of protein families, we compared the repertoire of IPRs of *R. mackenziei* against closely related black yeast species (*C. carrionii*, *C. bantiana*, *C. psammophila*, *E. dermatitidis*, and *E. xenobiotica*), and members of the orders Eurotiales (*Aspergillus fumigatus*, and *A. nidulans*) and Onygenales (*Paracoccidioides brasiliensis*, *Trichophyton rubrum*, and *Coccidioides immitis*). We confirmed the expansion of ADH-related domains (IPR013149 and IPR013154), CYP domains (IPR001128), and a trichothecene efflux pump domain (IPR010573) previously determined using a stochastic model of gene birth and death to estimate the evolution of gene family size (Teixeira *et al.* 2017). Additionally, the following domains were significantly more common in *R. mackenziei* and in other black yeasts: MFS transporters (IPR011701 and IPR020846) and sugar-associated transporters (IPR003663 and IPR005829), polyketide synthase (IPR020843), fungal transcription factor (IPR007219), and protein phosphorylation-related domains (Table S9).

The list of contractions in *R. mackenziei* includes the glucose/ribitol dehydrogenase (IPR002347), the CYP E-class (IPR002401), and the G-protein β WD-40 repeat (IPR020472) (Table S9).

### Pathogenicity-related genes

In total, 520 pathogenicity-related genes were found in *R. mackenziei*. In general, the species shared many potential virulence-associated homologs with the neurotropic fungus *Cryptococcus neoformans* (Table S10). Sequence analyses revealed that gene Z518_10204, with equivalent copies found in all three *R. mackenziei* strains examined, corresponded to the DEAD-box RNA helicase VAD1 (Panepinto *et al.* 2005). Protein Z518_10204 in *R. mackenziei* possessed the IPRs related to Vad1 protein—IPR001650, IPR014001, IPR014014, IPR027417 and IPR011545—supporting the functional annotation. Vad1 protein is believed to be involved in transcriptional regulation as well as in RNA stability, and plays an essential role in stress response, salt tolerance, and regulation of the virulence factor laccase (Panepinto *et al.* 2005). This protein is present in *Cr. neoformans* (75% identity to Z518_10204) and is expressed during infection of human brain (Panepinto *et al.* 2005). Other pathogenic species belonging to the order Onygenales, such as *Co. immitis* (83% identity) and *T. rubrum* (83% identity), possessed homologs to Z518_10204. Additionally, gene Z518_03960, which encodes a phosphoenolpyruvate carboxykinase (PCK1) and is a virulence factor dependent on Vad1, was found conserved in *R. mackenziei* compared with *Cr. neoformans* (64% identity).

Urease is another interesting factor shared by *R. mackenziei* and *C. neoformans*. This protein has been postulated as an important pathogenicity-related gene facilitating fungal central nervous system invasion (Olszewski *et al.* 2004; Singh *et al.* 2013). In general, black yeasts carry a single gene copy coding for urease, except *C. immunda*, which possesses two urease copies. Despite the presence of conserved IPRs specific to the urease accessory proteins (UreF, UreD, UreG, and NIC1), their amino-acid sequences are poorly conserved compared to *C. neoformans*. In contrast, Z518_09873, the *R. mackenziei* homolog of URE1, shares 62% of BLASTP identity with URE1 in *C. neoformans*. Accessory proteins are responsible for activating the urease apoenzyme and are thought to be involved in human brain infection (Singh *et al.* 2013).

*R. mackenziei* also harbors candidate pathogenicity orthologs in species of Eurotiales. The glucosamine-fructose-6-phosphate aminotransferase (GFA1) in *R. mackenziei* shares 88% of BLASTP identity with that in *A. fumigatus*. Gfa1 protein catalyzes the first step in the chitin biosynthesis pathway, and is believed to be essential and a potential drug target enzyme in *A. fumigatus* (Hu *et al.* 2007).

### Mitochondrial genomes

**Figure 6:**
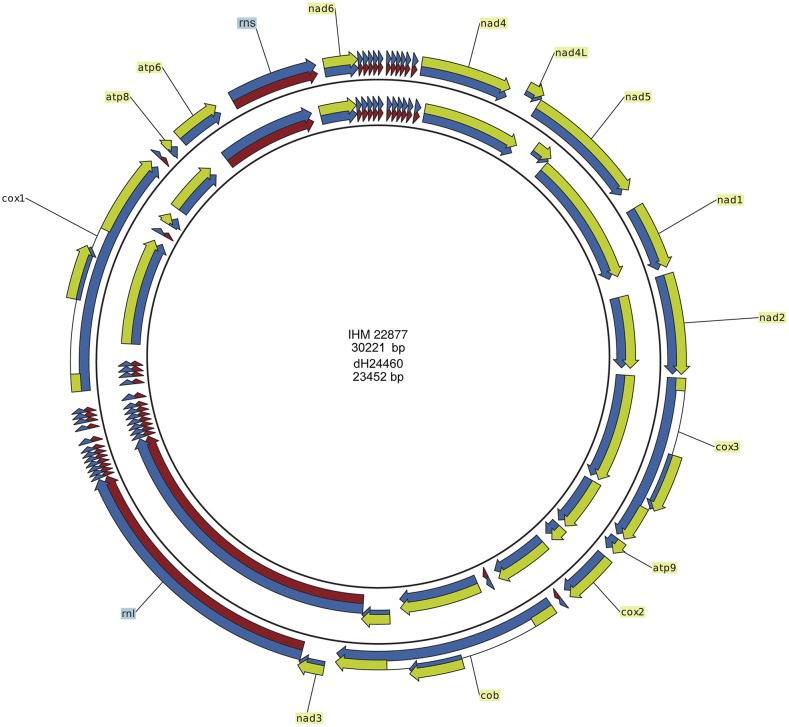
Mitochondrial genomes of *R. mackenziei* strains. IHM 22877 (inner circle) and dH24460 (outer circle). Blue arrows represent genes and yellow arrows are protein coding sequences. Red arrows indicate rRNA or tRNA coding sequences.

Contrary to nuclear DNA, the circular mitochondrial genome of *R. mackenziei* is AT-rich (∼26% G+C). It contains 14 conserved protein coding genes, two rRNA genes, and 25 tRNA genes, which code for all 20 amino acids (Figure 6). All genes are located on the same strand and > 88% of the mitochondrial genome is coding sequence. Strain dH24460 contains three introns inside protein coding genes (*cob*, *cox1*, and *cox3*), while IHM 22877 contains no introns. The introns are group I and contain homing endonuclease genes; two of them belong to the LAGLIDADG family and one to the GIY-YIG family (*cob*). Besides the intron difference there are minor (0.3%) sequence differences between the two strains. In total, 66 positions are variable, 24 of which are in intergenic and 42 are in coding sequences. The tRNA genes contain no variation, the rRNA genes contain 14 variable positions, and the protein-coding genes 28, but only five of them result in amino acid substitutions.

The intron inside *cob* has its closest hit with an intron at the same position inside the *cob* gene of *Fusarium oxysporum* with 37% query coverage, 8e−98 *E*-value, and 75% sequence identity. BLAST search of the NCBI database returned no results for the other two introns.

## Discussion

Our study catalogs the mutations present in two strains of the neurotropic fungus *R. mackenziei*. Additionally, SVs were assessed, revealing an unexpected amount of large inversions. Hypervariable sections were seen in the ends of scaffolds (probably indicating telomeric regions) composed by repetitive nucleotides, which are subject to higher levels of mutations in other fungi (Cuomo *et al.* 2007) (Figure 2). Repetitive DNA prediction supported the high incidence of these elements at both ends of the scaffolds. As demonstrated by SNPs and indels, genomic variation was more frequent in *R. mackenziei* strain dH24460 than in strain IHM 22877 (Figure 3). This finding suggests that this strain is more genetically divergent from the reference strain. In contrast, SV from the reference, such as long inversions, was greater in strain IHM22877.

Expanded protein families in *R. mackenziei*, such as the CYPs, ADHs, and ALDHs, are more susceptible to accumulating significant mutations, which can be important for the emergence of evolutionary novelties or may have possible deleterious effects (Table S2). The notorious abundance of CYPs, ADHs, and ALDHs in the genomes of black yeasts is partially explained by gene duplication events, occurring in the common ancestor of these organisms, by which new genes with similar functions were copied (Teixeira *et al.* 2017). Redundantly, we showed that there are significant mutations found in the paralogous gene sets. Taking the results together, we propose two scenarios to explain the accumulation of significant mutations in genes originated by duplication in *R. mackenziei*. First, the redundant copy became a nonfunctional pseudogene due to deleterious mutations, and second, these enzymes could be under positive selection driving niche-specific adaptation via neofunctionalization, although no evidence of functional divergence via neofunctionalization has ever been experimentally detected in black yeasts. Further analyses are required to determine whether gene duplication is a major force in the evolution of black yeasts.

Sequence comparisons with the best-studied neurotropic fungus, *Cr. neoformans*, provided important insights into genes and possible mechanisms involved in brain infection. Although pathologies caused by *R. mackenziei* (brain abscess), *C. neoformans*, and *C. gattii* [meningoencephalitis and propensity of *C. gattii* to form cryptococcomas (Sorrell 2001)] differ significantly, genes conserved in both species might explain common strategies to invade the host. For instance, the presence of urease, an enzyme used to scavenge for nitrogen in the environment that is often associated with fungal central nervous system invasion (Olszewski *et al.* 2004; Singh *et al.* 2013).

*R. mackenziei* possesses a large amount of proteins that are highly conserved across black yeasts. It is worth mentioning that the majority of these homologs are shared with recognized alkylbenzene-growing fungi, such as *E. xenobiotica* and *C. immunda* (De Hoog *et al.* 2006; Zhao *et al.* 2010), as well as with the neurotropic black fungi *C. bantiana* and *E. dermatitidis*. Indeed, a link between hydrocarbon assimilation and fungal neurotropism has been suggested (Prenafeta-Boldú *et al.* 2006). We see an overlap between pathways used to obtain nutrients and confer fungal resistance to extreme environmental conditions, and the potential usage of them in the host, such as the phenylalanine degradation pathway via phenylacetate and homogentisate found in *R. mackenziei*. Of note, phenylalanine is a precursor of tyrosine, which in turn is used for the biosynthesis of the monoamine neurotransmitters dopamine, norepinephrine (noradrenaline), and epinephrine (adrenaline), in addition to the formation of melanin and neuromelanin (a brain-protective dark pigment synthesized from L-dopamine). Phenylacetate is also derived from environmental contaminants such as styrene and ethylbenzene (Teufel *et al.* 2010; Fuchs *et al.* 2011). We also demonstrated that *R. mackenziei* possesses genes that might encode all enzymes involved in the toluene and styrene degradation pathways. The intermediate compounds produced during toluene assimilation, for instance protocatechuic acid (along with other structurally related compounds), are also abundant in the mammalian central nervous system and might serve as an important carbon source for *R. mackenziei* (Swartz *et al.* 1990).

Despite the fact that the environmental habitat of *R. mackenziei* is unknown, the genomic similarities between species isolated from habitats contaminated with aromatic hydrocarbons, especially with relatively mobile and bioavailable alkylbenzenes like toluene, ethylbenzene, xylene, and styrene, suggests that oil-polluted desert soil might be a potential reservoir of *R. mackenziei*. Toluene is a common chemical in oil and particularly in gasoline distillates, where it comprises ≤7% of the gasoline mass (Frysinger *et al.* 1999). Hydrocarbon-impacted soils provide a complex environment with diverse substrates, including short- and long-chain alkanes, monoaromatic and polycyclic aromatic hydrocarbons, etc., in which relatively few resistant organisms are able to thrive. Such an environment might give *R. mackenziei* a competitive advantage under the prevailing climatic conditions of the arid, oil-rich countries of the Middle East. In this study, three new members of the CYP52 family were identified. CYP52 genes are involved in fatty acid metabolism in the yeasts *Candida maltosa* and *Candida. tropicalis* (Seghezzi *et al.* 1992; Ohkuma *et al.* 1995). In addition, the CYP52 family is a key enzyme for the primary hydroxylation of n-alkanes (Huang *et al.* 2014), an acyclic saturated hydrocarbon widely found in petroleum (crude oil).

The role of the assimilation of toxic monoaromatic hydrocarbons by black yeasts as an evolutionary adaptation has been a subject of debate. Many *Exophiala* species are considered to be extremotolerant because of their selective advantage in environments that are enriched for hydrocarbon pollutants and heavy metals. Opportunistic pathogenicity of these fungi may be enhanced by extremetolerance (Zhao *et al.* 2010; Isola *et al.* 2013). On the other hand, the importance and the potential function of nitrogen-containing compounds have not yet been assessed in this group of fungi. Our analyses suggest that gene duplication events could be an important strategy in *R. mackenziei* to increase gene dosage along with the diversification of gene function involved in the uptake and metabolism of nitrogenous compounds. These findings suggest that *R. mackenziei*, in the absence of easily degradable sources, may have adapted to tolerate and coassimilate a wide range of toxic nitrogen-containing compounds as an alternative source of nutrients. Genes associated with different mechanisms of osmotic stress adaptation were found in *R. mackenziei*, including those involved in the storage of neutral and low-molecular-weight compounds, such as trehalose and choline. Interestingly, the acetylated derivative of choline, acetylcholine, is an indispensable neurotransmitter in higher animals that is widely found in the central and peripheral nervous systems (Picciotto *et al.* 2012).

Analysis of the secretome of *R. mackenziei* revealed the presence of 216 proteins that might be secreted via the classical endoplasmic reticulum/Golgi-dependent secretion pathway. Secretory proteins comprise a variety of enzymes involved in fungal pathogenicity and nutrient acquisition such as peptidases, laccases, tyrosinases, CAZY, and monooxygenases. Despite the high abundance of CYP genes in the genome of *R. mackenziei*, only CYP567 seems to be secreted. Indeed, P450s are intracellular or membrane-bound monooxygenases that have rarely been reported in outside the cell (Matsuzaki and Wariishi 2004; Ullrich and Hofrichter 2007). The amount of secretory laccases in *R. mackenziei* is consistent with what was recently described for black yeasts of the genus *Fonsecaea* (Moreno *et al.* 2017). Laccases may catalyze the oxidation of phenolic compounds and aromatic amines (Peng *et al.* 2015). The most abundant secreted peptidases belong to the S10 family, according to MEROPS (a proteolytic enzyme database), which contains a variable set of carboxypeptidases. Carboxypeptidase S10 was earlier described in the dermatophyte *T. rubrum* and was considered as a virulence factor (Zaugg *et al.* 2008). Moreover, peptidases of the families A01 (pepsin), S09 (serine-dependent peptidases), and S33 (exopeptidases) were found in multiple copies in the repertory of proteins with secreted proteolytic activity. Thus, we speculate that these secreted proteases are important for the virulence of *R. mackenziei*, allowing this fungus to grow on the nitrogenous substrates during infection.

The evolution of true mammal pathogens, such as members of the order Onygenales (Sharpton *et al.* 2009), is often associated with gene family loss leading to pathways lacking enzymes. Conversely, *R. mackenziei* possesses a wide arsenal of genes encoding enzymes for nutrient acquisition and primary metabolism (*e.g.*, nitrogen metabolism, carbohydrate metabolism, and fatty acid metabolism). Overall, the pathway collection in this fungus resembles that of black yeast that are able to assimilate alkylbenzene hydrocarbons. These results confirm that, unlike other true pathogens, *R. mackenziei* is a versatile fungus, with several pathways to sequester carbon from a wide range of nutrient substrates from the environment, possibly suggesting an oligotrophic lifestyle. In this respect, the fungus differs from true pathogens that need to extract nutrients from their hosts. Opportunism may be explained by the occurrence of virulence factors that have other roles in the natural life cycle of the fungus, such as the presence of a glyoxylate cycle pathway, different strategies for acquiring iron, toxin neutralization, and secondary metabolism (Teixeira *et al.* 2017). Key enzymes of the glyoxylate cycle are highly expressed during infection, possibly in response to conditions of starvation, *e.g.*, in the phagolysosome during phagocytosis (Lorenz and Fink 2001). However, recent publications suggest that the glyoxylate cycle might not be required for virulence in *Cr. neoformans* (Rude *et al.* 2002) and other fungi (Schobel *et al.* 2007), which opens up a debate on its clinical significance. Utilization of C_2_ compounds via the glyoxylate shunt might also be a nutritional strategy adopted by oligotrophic black yeasts to survive in nutrient-depleted environments, avoiding loss of carbons as CO_2_ due to the decarboxylation steps in the tricarboxylic acid cycle.

## Supplementary Material

Supplemental material is available online at www.g3journal.org/lookup/suppl/doi:10.1534/g3.117.300421/-/DC1.

## References

[bib1] Arzanlou M. Groenewald J. Z. Gams W. Braun U. Shin H. D., 2007 Phylogenetic and morphotaxonomic revision of *Ramichloridium* and allied genera. Stud. Mycol. 58: 57–93. 18490996 10.3114/sim.2007.58.03 PMC2104745

[bib2] Badali H. de Hoog G. S. Curfs-Breuker I. Meis J. F., 2010 *In vitro* activities of antifungal drugs against *Rhinocladiella mackenziei*, an agent of fatal brain infection. J. Antimicrob. Chemother. 65: 175–177. 19854862 10.1093/jac/dkp390

[bib3] Bankevich A. Nurk S. Antipov D. Gurevich A. A. Dvorkin M., 2012 SPAdes: a new genome assembly algorithm and its applications to single-cell sequencing. J. Comput. Biol. 19: 455–477. 22506599 10.1089/cmb.2012.0021 PMC3342519

[bib4] Blasi B. Tafer H. Kustor C. Poyntner C. Lopandic K., 2017 Genomic and transcriptomic analysis of the toluene degrading black yeast Cladophialophora immunda. Sci. Rep. 7: 11436. 28900256 10.1038/s41598-017-11807-8 PMC5595782

[bib5] Blomberg A. Adler L., 1992 Physiology of osmotolerance in fungi. Adv. Microb. Physiol. 33: 145–212.10.1016/S0065-2911(08)60217-9 1636508 10.1016/s0065-2911(08)60217-9

[bib6] Bolger A. M. Lohse M. Usadel B., 2014 Trimmomatic: a flexible trimmer for Illumina sequence data. Bioinformatics 30: 2114–2120. 24695404 10.1093/bioinformatics/btu170 PMC4103590

[bib7] Brankovics B. Z. H. van Diepeningen A. D. van der Lee T. A. J. Waalwijk C. de Hoog G. S., 2016 GRAbB: selective assembly of genomic regions, a new niche for genomic research. PLOS Comput. Biol. 12: e1004753. 27308864 10.1371/journal.pcbi.1004753 PMC4911045

[bib8] Chen K. Wallis J. W. McLellan M. D. Larson D. E. Kalicki J. M., 2009 BreakDancer: an algorithm for high-resolution mapping of genomic structural variation. Nat. Methods 6: 677–681. 19668202 10.1038/nmeth.1363 PMC3661775

[bib9] Chen W. Lee M. K. Jefcoate C. Kim S. C. Chen F., 2014 Fungal cytochrome p450 monooxygenases: their distribution, structure, functions, family expansion, and evolutionary origin. Genome Biol. Evol. 6: 1620–1634. 24966179 10.1093/gbe/evu132 PMC4122930

[bib10] Chen Z. Martinez D. A. Gujja S. Sykes S. M. Zeng Q., 2014 Comparative genomic and transcriptomic analysis of *Wangiella dermatitidis*, a major cause of phaeohyphomycosis and a model black yeast human pathogen. G3 (Bethesda) 4: 561–578. 24496724 10.1534/g3.113.009241 PMC4059230

[bib11] Choi Y. Sims G. E. Murphy S. Miller J. R. Chan A. P., 2012 Predicting the functional effect of amino acid substitutions and indels. PLoS One 7: e46688. 23056405 10.1371/journal.pone.0046688 PMC3466303

[bib12] Crešnar B. Petrič S., 2011 Cytochrome P450 enzymes in the fungal kingdom. Biochim. Biophys. Acta 1814: 29–35. 20619366 10.1016/j.bbapap.2010.06.020

[bib13] Cristini A. Garcia-Hermoso D. Celard M. Albrand G. Lortholary O., 2010 Cerebral phaeohyphomycosis caused by *Rhinocladiella mackenziei* in a woman native to Afghanistan. J. Clin. Microbiol. 48: 3451–3454. 20592148 10.1128/JCM.00924-10 PMC2937739

[bib14] Cuomo C. A. Guldener U. Xu J. R. Trail F. Turgeon B. G., 2007 The *Fusarium graminearum* genome reveals a link between localized polymorphism and pathogen specialization. Science 317: 1400–1402. 17823352 10.1126/science.1143708

[bib15] de Hoog G. S. Guarro J. Gené J. Figueras M. J., 2000 *Atlas of Clinical Fungi*. Centraalbureau Voor Schimmelcultures, Utrecht, The Netherlands, Universitat Rovira i Virgili, Reus, Spain.

[bib16] De Hoog G. S. Zeng J. S. Harrak M. J. Sutton D. A., 2006 *Exophiala xenobiotica* sp. nov., an opportunistic black yeast inhabiting environments rich in hydrocarbons. Antonie van Leeuwenhoek 90: 257–268. 16897561 10.1007/s10482-006-9080-z

[bib17] Faber B. W. van Gorcom R. F. Duine J. A., 2001 Purification and characterization of benzoate-*para*-hydroxylase, a cytochrome P450 (CYP53A1), from *Aspergillus niger*. Arch. Biochem. Biophys. 394: 245–254. 11594739 10.1006/abbi.2001.2534

[bib18] Frysinger G. S. Gaines R. B. Jr L. E. B., 1999 Quantitative determination of BTEX and total aromatic compounds in gasoline by comprehensive two-dimensional gas chromatography (GC×GC). J. High Resolut. Chromatogr. 22: 195–200.

[bib19] Fuchs G. Boll M. Heider J., 2011 Microbial degradation of aromatic compounds - from one strategy to four. Nat. Rev. Microbiol. 9: 803–816. 21963803 10.1038/nrmicro2652

[bib20] Ghoneim D. H. Myers J. R. Tuttle E. Paciorkowski A. R., 2014 Comparison of insertion/deletion calling algorithms on human next-generation sequencing data. BMC Res. Notes 7: 864. 25435282 10.1186/1756-0500-7-864 PMC4265454

[bib21] Horré R. de Hoog G., 1999 Primary cerebral infections by melanized fungi: a review. Stud. Mycol. 43: 176–193.

[bib22] Horton P. Park K. J. Obayashi T. Fujita N. Harada H., 2007 WoLF PSORT: protein localization predictor. Nucleic Acids Res. 35: W585–W587. 17517783 10.1093/nar/gkm259 PMC1933216

[bib23] Hu W. Sillaots S. Lemieux S. Davison J. Kauffman S., 2007 Essential gene identification and drug target prioritization in Aspergillus fumigatus. PLoS Pathog. 3: e24. 17352532 10.1371/journal.ppat.0030024 PMC1817658

[bib24] Huang F. C. Peter A. Schwab W., 2014 Expression and characterization of *CYP52* genes involved in the biosynthesis of sophorolipid and alkane metabolism from *Starmerella bombicola*. Appl. Environ. Microbiol. 80: 766–776. 24242247 10.1128/AEM.02886-13 PMC3911089

[bib25] Isola D. Selbmann L. de Hoog G. S. Fenice M. Onofri S., 2013 Isolation and screening of black fungi as degraders of volatile aromatic hydrocarbons. Mycopathologia 175: 369–379. 23475324 10.1007/s11046-013-9635-2

[bib26] Jacobson E. S., 2000 Pathogenic roles for fungal melanins. Clin. Microbiol. Rev. 13: 708–717. 11023965 10.1128/cmr.13.4.708-717.2000 PMC88958

[bib27] Jawallapersand P. Mashele S. S. Kovacic L. Stojan J. Komel R., 2014 Cytochrome P450 monooxygenase CYP53 family in fungi: comparative structural and evolutionary analysis and its role as a common alternative anti-fungal drug target. PLoS One 9: e107209. 25222113 10.1371/journal.pone.0107209 PMC4164535

[bib28] Kall L. Krogh A. Sonnhammer E. L., 2007 Advantages of combined transmembrane topology and signal peptide prediction–the Phobius web server. Nucleic Acids Res. 35: W429–W432. 17483518 10.1093/nar/gkm256 PMC1933244

[bib29] Kornberg H. L., 1966 The role and control of the glyoxylate cycle in Escherichia coli. Biochem. J. 99: 1–11. 10.1042/bj0990001 PMC1264949 5337756

[bib30] Li D. M. de Hoog G. S., 2009 Cerebral phaeohyphomycosis–a cure at what lengths? Lancet Infect. Dis. 9: 376–383. 19467477 10.1016/S1473-3099(09)70131-8

[bib31] Li H. Durbin R., 2009 Fast and accurate short read alignment with Burrows-Wheeler transform. Bioinformatics 25: 1754–1760. 19451168 10.1093/bioinformatics/btp324 PMC2705234

[bib32] Li L. Stoeckert C. J. Jr Roos D. S., 2003 OrthoMCL: identification of ortholog groups for eukaryotic genomes. Genome Res. 13: 2178–2189. 12952885 10.1101/gr.1224503 PMC403725

[bib33] Li X. Q. Guo B. L. Cai W. Y. Zhang J. M. Huang H. Q., 2016 The role of melanin pathways in extremotolerance and virulence of *Fonsecaea* revealed by *de novo* assembly transcriptomics using Illumina paired-end sequencing. Stud. Mycol. 83: 1–18. 27504027 10.1016/j.simyco.2016.02.001 PMC4969264

[bib34] Lorenz M. C. Fink G. R., 2001 The glyoxylate cycle is required for fungal virulence. Nature 412: 83–86. 11452311 10.1038/35083594

[bib35] Luykx D. M. Prenafeta-Boldu F. X. de Bont J. A., 2003 Toluene monooxygenase from the fungus *Cladosporium sphaerospermum*. Biochem. Biophys. Res. Commun. 312: 373–379. 14637148 10.1016/j.bbrc.2003.10.128

[bib36] Matsuzaki F. Wariishi H., 2004 Functional diversity of cytochrome P450s of the white-rot fungus *Phanerochaete chrysosporium*. Biochem. Biophys. Res. Commun. 324: 387–393. 15465031 10.1016/j.bbrc.2004.09.062

[bib37] Moreno L. F. Feng P. Weiss V. A. Vicente V. A. Stielow J. B., 2017 Phylogenomic analyses reveal the diversity of laccase-coding genes in *Fonsecaea* genomes. PLoS One 12: e0171291. 28187150 10.1371/journal.pone.0171291 PMC5302831

[bib38] Moriya Y. Itoh M. Okuda S. Yoshizawa A. C. Kanehisa M., 2007 KAAS: an automatic genome annotation and pathway reconstruction server. Nucleic Acids Res. 35: W182–W185. 17526522 10.1093/nar/gkm321 PMC1933193

[bib39] Nelson D. R., 1998 Metazoan cytochrome P450 evolution. Comp. Biochem. Physiol. C Pharmacol. Toxicol. Endocrinol. 121: 15–22. 9972448 10.1016/s0742-8413(98)10027-0

[bib40] Nikawa J. Hosaka K. Tsukagoshi Y. Yamashita S., 1990 Primary structure of the yeast choline transport gene and regulation of its expression. J. Biol. Chem. 265: 15996–16003. 2203793

[bib41] Ohkuma M. Muraoka S. Tanimoto T. Fujii M. Ohta A., 1995 *CYP52* (cytochrome P450alk) multigene family in *Candida maltosa*: identification and characterization of eight members. DNA Cell Biol. 14: 163–173. 7865134 10.1089/dna.1995.14.163

[bib42] Olszewski M. A. Noverr M. C. Chen G. H. Toews G. B. Cox G. M., 2004 Urease expression by *Cryptococcus neoformans* promotes microvascular sequestration, thereby enhancing central nervous system invasion. Am. J. Pathol. 164: 1761–1771. 15111322 10.1016/S0002-9440(10)63734-0 PMC1615675

[bib43] Panepinto J. Liu L. Ramos J. Zhu X. Valyi-Nagy T., 2005 The DEAD-box RNA helicase Vad1 regulates multiple virulence-associated genes in *Cryptococcus neoformans*. J. Clin. Invest. 115: 632–641. 15765146 10.1172/JCI200523048 PMC1051994

[bib44] Paolo W. F. Jr Dadachova E. Mandal P. Casadevall A. Szaniszlo P. J., 2006 Effects of disrupting the polyketide synthase gene WdPKS1 in Wangiella [Exophiala] dermatitidis on melanin production and resistance to killing by antifungal compounds, enzymatic degradation, and extremes in temperature. BMC Microbiol. 6: 55. 16784529 10.1186/1471-2180-6-55 PMC1569847

[bib45] Parales R. E. Parales J. V. Pelletier D. A. Ditty J. L., 2008 Diversity of microbial toluene degradation pathways. Adv. Appl. Microbiol. 64: 1–73. 18485280 10.1016/S0065-2164(08)00401-2

[bib46] Park Y. I. Gander J. E., 1998 Choline derivatives involved in osmotolerance of *Penicillium fellutanum*. Appl. Environ. Microbiol. 64: 273–278. 16349488 10.1128/aem.64.1.273-278.1998 PMC124705

[bib47] Pavesi A. Conterio F. Bolchi A. Dieci G. Ottonello S., 1994 Identification of new eukaryotic tRNA genes in genomic DNA databases by a multistep weight matrix analysis of transcriptional control regions. Nucleic Acids Res. 22: 1247–1256. 8165140 10.1093/nar/22.7.1247 PMC523650

[bib48] Pedrini N. Ortiz-Urquiza A. Huarte-Bonnet C. Zhang S. Keyhani N. O., 2013 Targeting of insect epicuticular lipids by the entomopathogenic fungus *Beauveria bassiana*: hydrocarbon oxidation within the context of a host-pathogen interaction. Front. Microbiol. 4: 24. 23422735 10.3389/fmicb.2013.00024 PMC3573267

[bib49] Peng X. Yuan X. Z. Liu H. Zeng G. M. Chen X. H., 2015 Degradation of polycyclic aromatic hydrocarbons (PAHs) by laccase in rhamnolipid reversed micellar system. Appl. Biochem. Biotechnol. 176: 45–55. 25637508 10.1007/s12010-015-1508-3

[bib50] Petersen T. N. Brunak S. von Heijne G. Nielsen H., 2011 SignalP 4.0: discriminating signal peptides from transmembrane regions. Nat. Methods 8: 785–786. 21959131 10.1038/nmeth.1701

[bib51] Picciotto M. R. Higley M. J. Mineur Y. S., 2012 Acetylcholine as a neuromodulator: cholinergic signaling shapes nervous system function and behavior. Neuron 76: 116–129. 23040810 10.1016/j.neuron.2012.08.036 PMC3466476

[bib52] Podobnik B. Stojan J. Lah L. Krasevec N. Seliskar M., 2008 CYP53A15 of *Cochliobolus lunatus*, a target for natural antifungal compounds. J. Med. Chem. 51: 3480–3486. 18505250 10.1021/jm800030e

[bib53] Prenafeta-Boldú F. X. Summerbell R. Sybren de Hoog G., 2006 Fungi growing on aromatic hydrocarbons: biotechnology’s unexpected encounter with biohazard? FEMS Microbiol. Rev. 30: 109–130. 16438682 10.1111/j.1574-6976.2005.00007.x

[bib54] Rodriguez-Saiz M. Barredo J. L. Moreno M. A. Fernandez-Canon J. M. Penalva M. A., 2001 Reduced function of a phenylacetate-oxidizing cytochrome p450 caused strong genetic improvement in early phylogeny of penicillin-producing strains. J. Bacteriol. 183: 5465–5471. 11544206 10.1128/JB.183.19.5465-5471.2001 PMC95435

[bib55] Rude T. H. Toffaletti D. L. Cox G. M. Perfect J. R., 2002 Relationship of the glyoxylate pathway to the pathogenesis of *Cryptococcus neoformans*. Infect. Immun. 70: 5684–5694. 12228298 10.1128/IAI.70.10.5684-5694.2002 PMC128360

[bib56] Schobel F. Ibrahim-Granet O. Ave P. Latge J. P. Brakhage A. A., 2007 *Aspergillus fumigatus* does not require fatty acid metabolism via isocitrate lyase for development of invasive aspergillosis. Infect. Immun. 75: 1237–1244. 17178786 10.1128/IAI.01416-06 PMC1828595

[bib57] Seghezzi W. Meili C. Ruffiner R. Kuenzi R. Sanglard D., 1992 Identification and characterization of additional members of the cytochrome P450 multigene family *CYP52* of *Candida tropicalis*. DNA Cell Biol. 11: 767–780. 1457045 10.1089/dna.1992.11.767

[bib58] Seyedmousavi S. Netea M. G. Mouton J. W. Melchers W. J. Verweij P. E., 2014 Black yeasts and their filamentous relatives: principles of pathogenesis and host defense. Clin. Microbiol. Rev. 27: 527–542. 24982320 10.1128/CMR.00093-13 PMC4135901

[bib59] Sharpton T. J. Stajich J. E. Rounsley S. D. Gardner M. J. Wortman J. R., 2009 Comparative genomic analyses of the human fungal pathogens *Coccidioides* and their relatives. Genome Res. 19: 1722–1731. 19717792 10.1101/gr.087551.108 PMC2765278

[bib60] Singh A. Panting R. J. Varma A. Saijo T. Waldron K. J., 2013 Factors required for activation of urease as a virulence determinant in *Cryptococcus neoformans*. MBio 4: e00220-13. 23653445 10.1128/mBio.00220-13 PMC3663189

[bib61] Slater G. S. Birney E., 2005 Automated generation of heuristics for biological sequence comparison. BMC Bioinformatics 6: 31. 15713233 10.1186/1471-2105-6-31 PMC553969

[bib62] Sonnhammer E. L. von Heijne G. Krogh A., 1998 A hidden Markov model for predicting transmembrane helices in protein sequences. Proc. Int. Conf. Intell. Syst. Mol. Biol. 6: 175–182. 9783223

[bib63] Sorrell T. C., 2001 *Cryptococcus neoformans* variety *gattii*. Med. Mycol. 39: 155–168. 11346263

[bib64] Swartz K. J. Matson W. R. MacGarvey U. Ryan E. A. Beal M. F., 1990 Measurement of kynurenic acid in mammalian brain extracts and cerebrospinal fluid by high-performance liquid chromatography with fluorometric and coulometric electrode array detection. Anal. Biochem. 185: 363–376. 2339792 10.1016/0003-2697(90)90309-w

[bib65] Teixeira M. M. Moreno L. F. Stielow B. J. Muszewska A. Hainaut M., 2017 Exploring the genomic diversity of black yeasts and relatives (Chaetothyriales, Ascomycota). Stud. Mycol. 86: 1–28. 28348446 10.1016/j.simyco.2017.01.001 PMC5358931

[bib66] Teufel R. Mascaraque V. Ismail W. Voss M. Perera J., 2010 Bacterial phenylalanine and phenylacetate catabolic pathway revealed. Proc. Natl. Acad. Sci. USA 107: 14390–14395. 20660314 10.1073/pnas.1005399107 PMC2922514

[bib67] Ullrich R. Hofrichter M., 2007 Enzymatic hydroxylation of aromatic compounds. Cell. Mol. Life Sci. 64: 271–293. 17221166 10.1007/s00018-007-6362-1 PMC11138414

[bib68] Van der Auwera G. A. Carneiro M. O. Hartl C. Poplin R. Del Angel G., 2013 From FastQ data to high confidence variant calls: the Genome Analysis Toolkit best practices pipeline. Curr. Protoc. Bioinformatics 43: 11.10.1–11.10.33. 25431634 10.1002/0471250953.bi1110s43 PMC4243306

[bib69] van Gelder C. W. Flurkey W. H. Wichers H. J., 1997 Sequence and structural features of plant and fungal tyrosinases. Phytochemistry 45: 1309–1323. 9237394 10.1016/s0031-9422(97)00186-6

[bib70] Walker B. J. Abeel T. Shea T. Priest M. Abouelliel A., 2014 Pilon: an integrated tool for comprehensive microbial variant detection and genome assembly improvement. PLoS One 9: e112963. 25409509 10.1371/journal.pone.0112963 PMC4237348

[bib71] Winnenburg R. Baldwin T. K. Urban M. Rawlings C. Kohler J., 2006 PHI-base: a new database for pathogen host interactions. Nucleic Acids Res. 34: D459–D464. 16381911 10.1093/nar/gkj047 PMC1347410

[bib72] Ye K. Schulz M. H. Long Q. Apweiler R. Ning Z., 2009 Pindel: a pattern growth approach to detect break points of large deletions and medium sized insertions from paired-end short reads. Bioinformatics 25: 2865–2871. 19561018 10.1093/bioinformatics/btp394 PMC2781750

[bib73] Zaugg C. Jousson O. Lechenne B. Staib P. Monod M., 2008 *Trichophyton rubrum* secreted and membrane-associated carboxypeptidases. Int. J. Med. Microbiol. 298: 669–682. 18222721 10.1016/j.ijmm.2007.11.005

[bib74] Zhao J. Zeng J. de Hoog G. S. Attili-Angelis D. Prenafeta-Boldú F. X., 2010 Isolation and identification of black yeasts by enrichment on atmospheres of monoaromatic hydrocarbons. Microb. Ecol. 60: 149–156. 20333373 10.1007/s00248-010-9651-4 PMC2917551

