# Supplementary figures and images for: Genomic Understanding of an Infectious Brain Disease from the Desert
Source: G3 (Bethesda). 2018 Jan 11;8(3):909–22. doi: 10.1534/g3.117.300421 (PMC5844311; doi:10.1534/g3.117.300421)

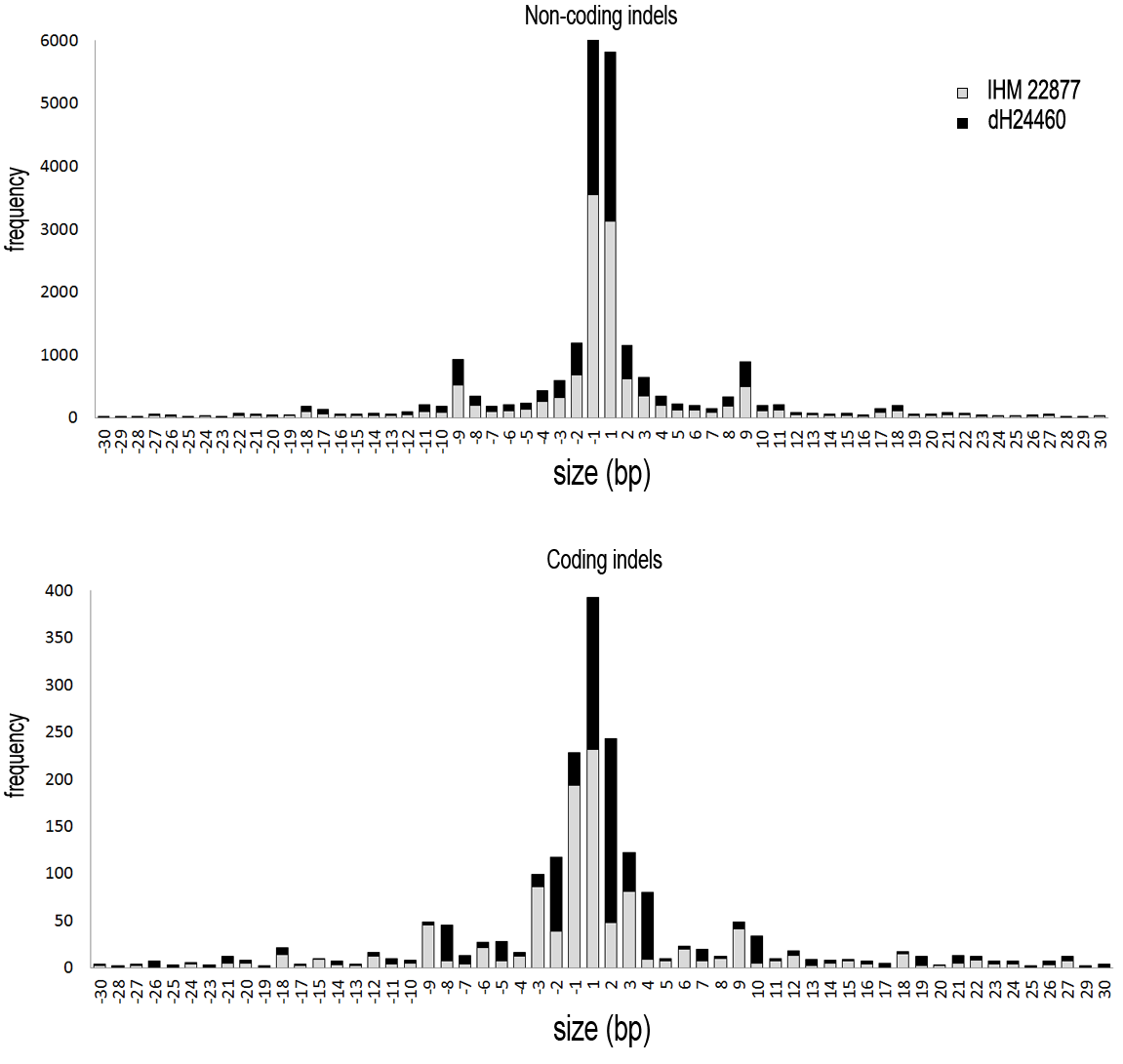


**Figure S1** – Frequency and size of indels in coding and non-coding regions.

Supplement: Supplementary file 1 [file 909FigureS1.docx]
